# Increased expression of MUC3A is associated with poor prognosis in localized clear-cell renal cell carcinoma

**DOI:** 10.18632/oncotarget.10312

**Published:** 2016-06-27

**Authors:** Tian Niu, Yidong Liu, Yuan Zhang, Qiang Fu, Zheng Liu, Zewei Wang, Hangcheng Fu, Jiejie Xu, Kun Liu

**Affiliations:** ^1^ Department of Ophthalmology, Shanghai General Hospital, Shanghai Jiaotong University School of Medicine, Shanghai 200080, China; ^2^ Department of Biochemistry and Molecular Biology, School of Basic Medical Sciences, Fudan University, Shanghai 200032, China

**Keywords:** clear-cell renal cell carcinoma, MUC3A, overall survival, recurrence-free survival, prognostic biomarker

## Abstract

MUC3A is a membrane-associated mucin that recent evidence reveals the role of MUC3A in pathogenesis and progression of cancers. To evaluate the association between MUC3A expression with overall survival (OS) and recurrence-free survival (RFS) in patients with localized clear-cell renal cell carcinoma (ccRCC), we retrospectively detected MUC3A expression in samples of 384 postoperative localized ccRCC patients by immunohistochemistry. Median follow-up was 73 months (range: 42 – 74 mo). Overall, 41 patients died, 47 experienced recurrence. High MUC3A expression occurred in 45.8% of localized ccRCC cases, which was significantly associated with high pT-stage, high Fuhrman grade, high frequency of necrosis and LVI, and increased risk of recurrence and death (Logrank test *P* < 0.001 and *P* < 0.001, respectively). By multivariate analysis, MUC3A expression was confirmed as an adverse independent prognostic factor for OS and RFS. The prognostic accuracy of UISS, SSIGN, Leibovich models was significantly increased when MUC3A expression was integrated. Meanwhile, MUC3A was enrolled into a newly built nomogram with other factors selected by multivariate analysis. Calibration curves revealed optimal consistency between observations and prognosis. In conclusion, high MUC3A expression is an adverse prognostic biomarker for OS and RFS in postoperative localized ccRCC patients.

## INTRODUCTION

Renal cell carcinoma (RCC) was ranked the seventh most common tumor worldly, with over 350 000 new cases in 2013 and 140000 death cases worldwide per year [1]. The increased incidence of RCC induced continuously raising of mortality rate among last three decades [2]. As the most predominant subtype, clear cell RCC (ccRCC) accounted nearly 70–75% in the kidney cancer [3]. Even the ccRCC patients of similar or same subtype usually performed complex and unpredictable course of disease and clinical outcomes. Another challenge in management of RCC is that most localized RCC patients could be cured by surgical intervention, but the prognosis is poor when patients experience local recurrence or distant metastasis. The reason is that renal carcinoma is usually resistant to conventional chemotherapy and radiotherapy [4]. On the whole, numerous molecular researches in the past have improved substantial insight to the biology of ccRCC and are instituteing to impact clinical practice and thereby better patient outcomes. Due to the complexity of signal pathways in ccRCC, even more extensive biomarkers are required to improve diagnosis, therapy and postoperative management.

Mucins are extracellular high molecular with heavily O-glycosylate, which are produced by various epithelial cells and attached to threnonie or serine residues of mucin core protein backbone through O-glycosidic linkages. Mucins build a selective molecular barrier and act in morphogenetic signal transduction. Alterations with mucin expression or glycosylation can affect cell growth, differentiation, adhesion, invasion and immune surveillance [5]. There is increasing evidence between aberrant glycosylations or expression of mucin peptides and neoplasms, such as MUC1, MUC2, MUC5AC in pancreatic neoplasm; MUC1, MUC4 in Gastrointestinal neoplasms [6]. Presumably, the process of natural selection with growing tumour-cell populations produces cells that express novel modes and combinations of mucins, which protects these tumour cells when they are invading and metastasizing [5]. All these studies may bring a bright prospect in pathogenesis, treatment and prognosis that between mucins and neoplasms.

Mucins are classified into 11 membrane-bound mucins and 7 secreted mucins. Analogous to MUC3A, MUC1, MUC3B, MUC4, MUC12, MUC13, MUC15, MUC16, MUC17, MUC20 and MUC21 are all membrane-bound mucins [6]. Membrane-bound mucins shares conserved domains, such as Sea urchin sperm protein Enterokinase and Agrin (SEA) domains or epidermal growth factor-like (EGF). Due to their localization and structure at the cell surface, they may participate in cell signaling, in cell–matrix and cell–cell interactions and in modulating biological properties under normal and pathological conditions [7].

MUC3A was mapped to a mucin cluster on chromosome 7q22 with tandem repeats of 17 amino acids, and categorized as a membrane-associated mucin [8]. On chromosomal locus 7q22, there are two different MUC3 genes were discovered that were named MUC3A and MUC3B, respectively. The researchers concluded that MUC3A is possibly the MUC3 gene originally described [9]. MUC3A includes two extracellular cysteine-rich epidermal growth factor-like domains that maybe implicated in cell proliferation through growth factors [8]. Poor prognosis with abnormal high MUC3A expression has been researched in breast, pancreatic, gastric, colorectal, appendiceal and prostate cancer [10-15]. Especially, there is a previous study using RT-PCR found that MUC3A was overexpressed in ccRCC at gene level, but had no statistically significant relationship with prognosis [4]. It may result from small size samples of twenty-six.

Here, whether the MUC3A play a role with carcinogenesis and progression in ccRCC is still unclear. Only few studies about association between MUC3A and clinical characteristics in ccRCC. In this manuscript, our goal is to evaluate the correlation between MUC3A expression and clinical characteristics, as well as the prognostic parameters in localized ccRCC. Additionally, we combined MUC3A expression with established prognostic models and create a new prognostic model by nomogram to assess the value of MUC3A in localized ccRCC.

## RESULTS

### MUC3A expression is association with clinicopathologic characteristics of localized ccRCC patients

**Figure 1:**
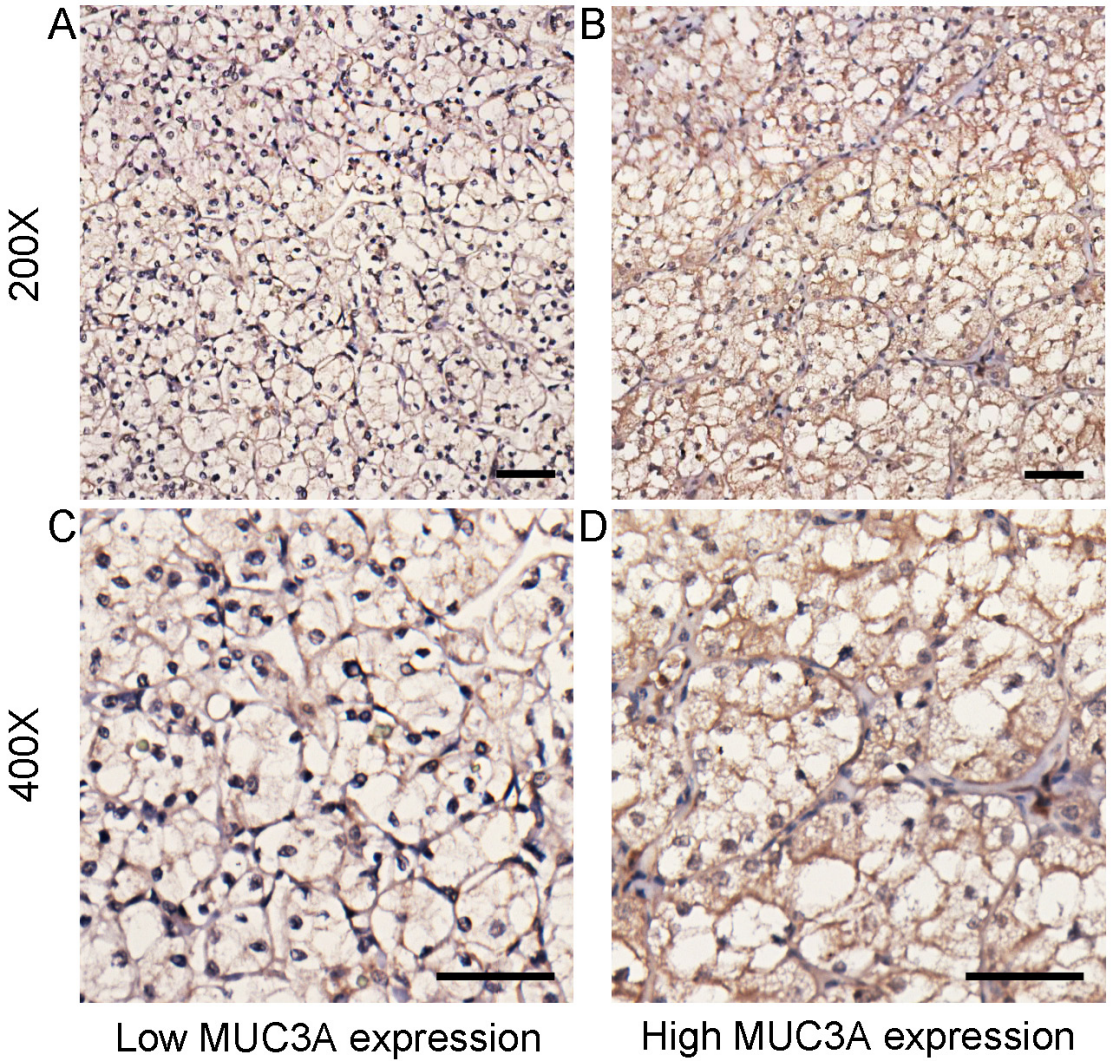
MUC3A expression in localized clear-cell renal cell carcinoma (ccRCC) tissues Representative MUC3A immunohistochemical (IHC) images of localized ccRCC tissues with low expression level **A.** and **C.** and high expression level **B.** and **D.** (original magnification x 200, x400). Scale bar: 50 μm.

With the presentation of Figure 1, variable intensity of immunohistochemical staining was observed in different specimens with score ranges from 0 to 196. We used score 90 to cut the 384 patients into “MUC3A low” group (n = 208) and “MUC3A high” group (n = 176). Median follow-up was 73 months (range: 42-74 mo). There were 41 (10.7%) patients died and 47 (12.2%) patients confirmed with tumor recurrence at last follow-up. Table 1 listed the association between MUC3A expression and characteristics of localized ccRCC patients. MUC3A expression was remarkably positively associated with pT-stage (*P* = 0.003), necrosis (*P* < 0.001), LVI (*P* = 0.005), and Fuhrman grade (*P* <0.001).

**Table 1:** Correlation between MUC3A expression andpatient characteristics

Characteristic	Patients (n=384)	MUC3A expression		P^a^
		Low (n=208)	High (n=176)	
Age at surgery (year)^b^	53.89 ± 14.91	54.17 ± 14.59	53.55 ±15.31	0.682
Gender				0.323
Female	111 (28.9%)	65	46	
Male	273 (71.1%)	143	130	
Tumor size (cm)^b^	4.15 ± 2.17	3.93 ± 1.99	4.41 ± 2.35	0.065
pT-stage				**0.003**
1	273 (71.1%)	163	110	
2	22 (5.7%)	10	12	
3	89 (23.2%)	35	54	
Necrosis				**<0.001**
Absent	309 (80.5%)	182	127	
Present	75 (19.5%)	26	49	
LVI				**0.005**
Absent	295 (76.8%)	172	123	
Present	89 (23.2%)	36	53	
Fuhrman grade				**<0.001**
1	66 (17.2%)	44	22	
2	189 (49.2%)	108	81	
3	89 (23.2%)	46	43	
4	40 (10.4%)	10	30	
ECOG-PS				0.121
0	323 (84.1%)	181	142	
≥1	61 (15.9%)	27	34	

LVI = lymphovascularinvasion; ECOG-PS = Eastern Cooperative Oncology Group performance status.

a *P*<0.05 is considered statistically significant.

b The results of continuous variables are presented as mean±SD (standard deviation).

### High MUC3A expression is associated poor prognosis of OS and RFS in patients with localized ccRCC

**Figure 2:**
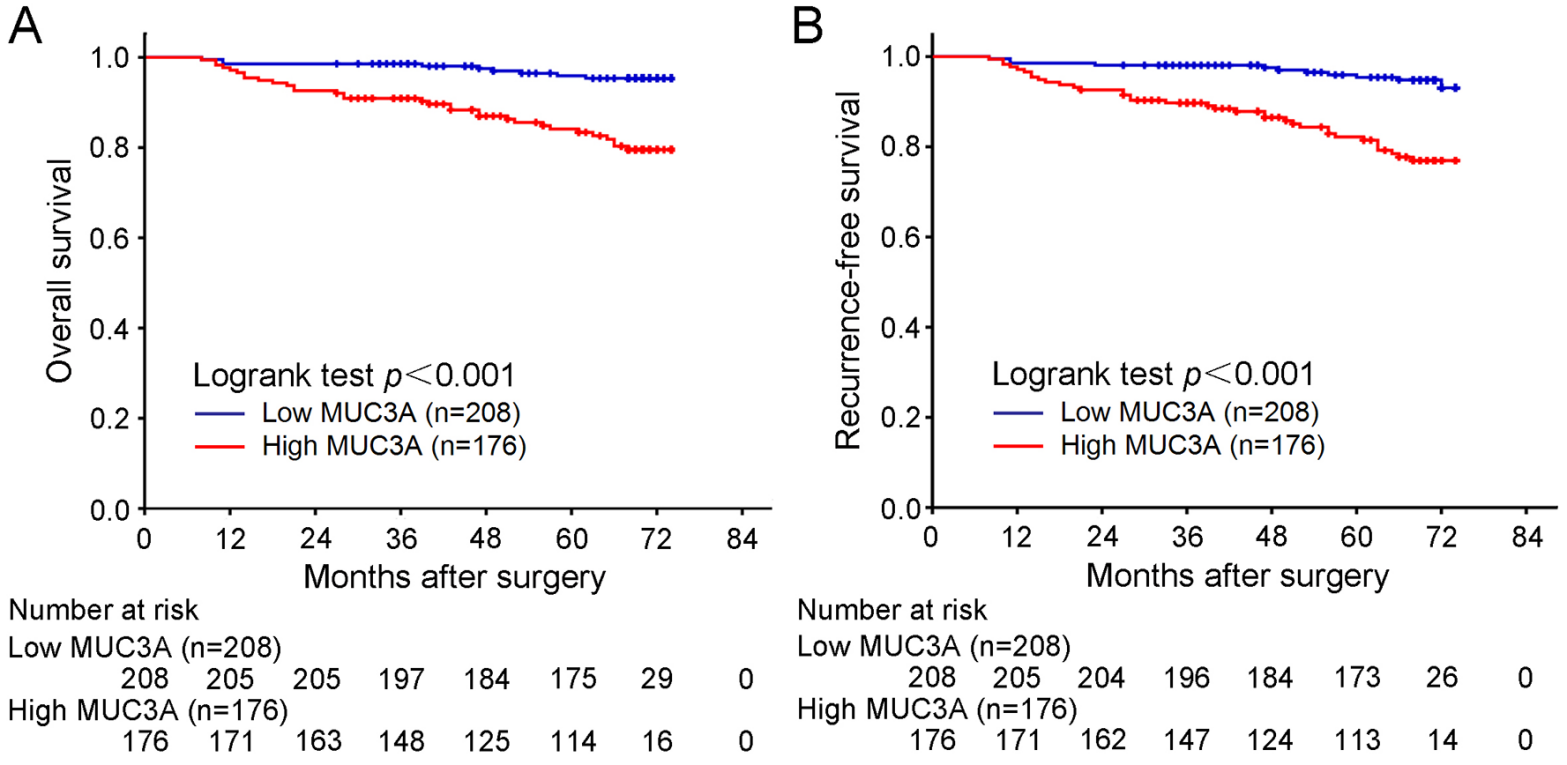
Overall survival (OS) and Recurrence-free survival (RFS) analysis of patients with localized ccRCC based on MUC3A expression Kaplan-Meier analysis of OS **A.** Kaplan-Meier analysis of RFS **B.** *P* value was calculated by log-rank test.

We used Kaplan-Meier method with log-rank test to analysis the relationship between MUC3A expression and clinical outcomes. The median follow-up was 73 months (range from 42-74 months). As shown in Figure 2A and Figure 2B, MUC3A expression was significantly related with OS (*P* < 0.001) and RFS (*P* < 0.001) of localized ccRCC patients. Those figures meant that higher MUC3A expression indicated earlier death and recurrence. Further subgroup analysis in patients stratified by T category or Fuhrman grade was perfomed to explore the effect of MUC3A expression on OS and RFS (Supplementary Figure S1, Supplementary Figure S2). As presented in the revised Supplementary Figure S1, T1 and T3 stages could be stratified by MUC3A expression in overall survival (T1 stage: P = 0.001, T3 stage: P = 0.046, respectively), as well as reccurrence-free survival (T1 stage: P < 0.001, T3 stage: P = 0.012, respectively). But T2 stage can't be stratified by MUC3A expression (overall survival: P =0.260, reccurrence-free survival: P = 0.763, respectively). As presented in the revised Supplementary Figure S2, only Fuhrman grade 2 could be stratified by MUC3A expression in overall survival (Fuhrman grade 1: P = 0.157, Fuhrman grade 2: P = 0.015, Fuhrman grade 3: P = 0.052, Fuhrman grade 4: P = 0.334, respectively). Meanwhile, Fuhrman grade 1 and grade 2 could be stratified by MUC3A expression in reccurence-free survival (Fuhrman grade 1: P = 0.047, Fuhrman grade 2: P = 0.037, Fuhrman grade 3: P = 0.115, Fuhrman grade 4: P = 0.088, respectively). Moreover, we compared RFS between radical nephrectomy cases and partical nephrectomy cases and found that both could be stratified by MUC3A expression (Supplementary Figure S3).

### High MUC3A expression is an identified poor prognostic indicator to localized ccRCC patients

To analysis the clinical significance of MUC3A expression for postoperative outcomes among localized ccRCC patients, univariate and multivariate analyses were used for OS and RFS. According to univariate Cox regression analysis, we selected several prognostic factors whose *P* value was < 0.05 in univariate analysis into multivariate Cox regression analysis. As shown in Table 2, All prognostic factors in multivariate analysis were independent and identified in OS. Furthermore, high MUC3A expression was found to be a identified negative prognostic factor for localized ccRCC patients in OS (HR, 2.509; 95 % CI, 1.136 to 5.539; *P* < 0.001) and RFS (HR, 2.658; 95 % CI, 1.287 to 5.489; *P* = 0.008). Meanwhile, tumor size (*P* < 0.001 and *P* < 0.001, respectively), necrosis (*P* = 0.005 and *P* < 0.001, respectively), LVI (*P* = 0.005 and *P* = 0.017, respectively), Fuhrman grade (*P* < 0.001 and *P* < 0.001, respectively) were also statistically identified factors affecting among localized ccRCC patients in both OS and RFS. Nevertheless, pT-stage (*P* = 0.018 and *P* = 0.095, respectively) and ECOG-PS (*P* = 0.033 and *P* = 0.053, respectively) were included for OS but excluded for RFS in the multivariate analysis.

**Table 2:** Univariate and multivariate cox regression analysis of overall survival and recurrence-free survival

Characteristic	Overall survival			Recurrence-free survival		
	Univariate analysis *P*^a^	Multivariate analysis		Univariate analysis *P*^a^	Multivariate analysis	
		Hazard Ratio (95% CI)	*P*^a^		Hazard Ratio (95% CI)	*P*^a^
Age at surgery (year)	0.969			0.431		
Gender	0.193			0.617		
Female						
Male						
Tumor size (cm)	**<0.001**	1.287 (1.108-1.494)	**0.001**	**<0.001**	1.324 (1.146-1.529)	**<0.001**
pT-stage	**<0.001**		**0.018**	**<0.001**		0.095
1		Reference			Reference	
2		2.561 (0.747-8.776)	0.135		1.861 (0.577-6.006)	0.299
3		3.485 (1.466-8.282)	**0.005**		2.449 (1.090-5.505)	**0.030**
Necrosis	**<0.001**		**0.005**	**<0.001**		**<0.001**
Absent		Reference			Reference	
Present		2.791 (1.375-5.667)			3.421 (1.793-6.526)	
LVI	**<0.001**		**0.005**	**<0.001**		**0.017**
Absent		Reference			Reference	
Present		2.868 (1.375-5.667)			2.291 (1.163-4.513)	
Fuhrman grade	**<0.001**		**<0.001**	**<0.001**		**<0.001**
1		Reference			Reference	
2		2.555 (0.330-19.774)	0.371		1.446 (0.321-6.526)	0.631
3		5.996 (0.753-47.738)	0.091		3.680 (0.806-16.796)	0.093
4		14.421 (1.824-114.016)	**0.011**		8.806 (1.942-39.927)	**0.005**
ECOG-PS	**<0.001**		**0.033**	**<0.001**		0.053
0		Reference			Reference	
≥1		2.198 (1.065-4.536)			1.956 (0.990-3.861)	
MUC3A	**<0.001**		**0.023**	**<0.001**		**0.008**
Low		Reference			Reference	
High		2.509 (1.136-5.539)			2.658 (1.287-5.489)	

CI = confidence interval; LVI = lymphovascular invasion; ECOG-PS = Eastern Cooperative Oncology Group performance status.

a *P*<0.05 is considered statistically significant.

### Extension of UISS, SSIGN and Leibovich prognostic models with MUC3A signature

In order to detect the prognosis precision of MUC3A with established prognostic models, we chose UISS, SSIGN and Leibovich prognostic models into our study. As showed in Table 3, the prognosis precision of OS was all improved from 0.723 to 0.781, from 0.768 to 0.830, from 0.820 to 0.859, respectively. Meanwhile, precision of RFS was enhanced from 0.724 to 0.779, from 0.756 to 0.812, from 0.815 to 0.847, respectively. The MUC3A signature only prognosis precision of OS and RFS were both 0.679.

**Table 3:** Comparison of the predictive accuracy of the prognostic models

Prognostic model	Overall survival		Recurrence-free survival	
	C-index	*P*	C-index	*P*
MUC3A signature	0.679		0.679	
UISS	0.723		0.724	
UISS + MUC3A signature	0.781	< 0.001	0.779	< 0.001
SSIGN	0.768		0.756	
SSIGN + MUC3A signature	0.830	0.002	0.812	0.004
Leibovich	0.820		0.815	
Leibovich +MUC3A signature	0.859	0.003	0.847	0.011

C-index Harrell's concordance index, UISS University of California Los Angeles Integrated Staging System, SSIGN stage, size, grade, and necrosis

### Prognostic nomogram for OS and RFS of patients with localized ccRCC

**Figure 3:**
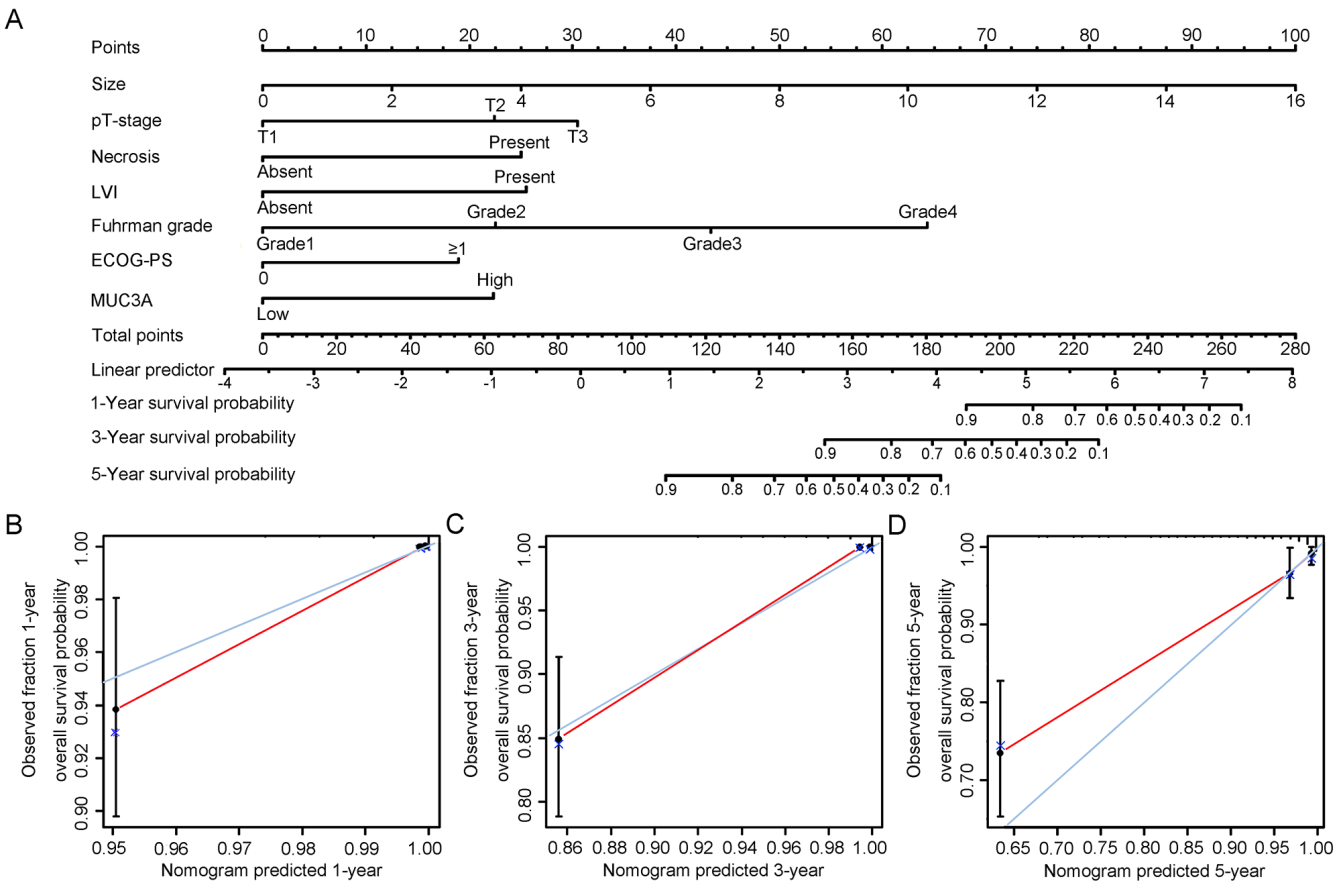
Nomogram and calibration plot for prognosis of OS in patients with localized ccRCC Postoperative prognostic nomogram of patients with localized ccRCC **A.** The calibration plots for overall survival at 1 years **B.** 3 years **C.** and 5 years **D.**

**Figure 4:**
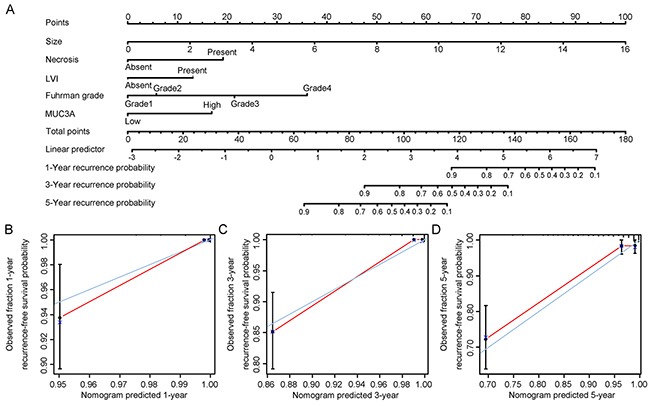
Nomogram and calibration plot for prognosis of RFS in patients with localized ccRCC Postoperative prognostic nomogram of patients with localized ccRCC **A.** The calibration plots for recurrence at 1 years **B.** 3 years **C.** and 5 years **D.**

We selected factors whose *P* value < 0.05 in multivariate Cox regression analysis to build a prognostic nomogram for OS and RFS (Figure 3A, Figure 4A). A well consistency of probability of OS and RFS at 1-, 3-, 5-year after surgery was shown by calibration plot (Figure 3B-3D, Figure 4B-4D).

## DISCUSSION

According to the results above, MUC3A expression has been proved as an adverse independent prognostic factor. Moreover, MUC3A expression can not only cooperate with well-established models to improve prognosis accuracy, but also participate in constructing nomogram with well effect. This work indicated that MUC3A may play an important role in oncogenesis and progression in localized ccRCC.

Although MUC3A had been proved as an independent prognosticator in localized ccRCC, its molecular mechanism in localized ccRCC is still uncertain. MUC3A expression is regulated at different levels. For instance, a tetrameric-branched peptide through a conserved TFLK motif and hypoxia through HIF-1α could molecularly induce elevated MUC3A expression [16, 17]. Thaher et al. demonstrated that cystic fibrosis transmembrane conductance regulator (CFTR) and MUC3A competed to bind the singtle PDZ domain on Golgi-associated PDZ and coiled-coil motif containing (GOPC), which regulated the relative levels of CFTR and MUC3A in turn [18]. Epigenetically, expression of MUC3A was impacted by promoter DNA hypomethylation [19]. MUC3A adjusted cell migration and apoptosis through tyrosine phosphorylation of two EGF cysteine-rich domains [20]. Similar to MUC1, SEA module at the C-terminal domain of MUC3A was critical for its autoproteolysis to impact migration and invasion by HER/ErbB2 phosphorylation [21]. In addition, MUC3A expression may regulated by PKC signal pathway and therefore modulated the invasive and metastatic properties in cancer cells [22]. In our study, the result that MUC3A expression was observably associated with pT-stage, necrosis, LVI, and Fuhrman grade may verify these basic researches. So combine previous studies with our result, we can hypothesis MUC3A play an import role in migration, invasion and malignant and result in poor prognosis of localized ccRCC patients with high MUC3A expression. In addition, MUC3A may become a therapeutic target. By inhibiting MUC3A, the rate of tumor evolvement maybe decreases and improves the effectiveness of existing therapeutics.

Consistent with other cancers types according to former studies, high MUC3A expression was identified as an adverse independent prognosticator for OS and RFS in localized ccRCC following surgery. Furthermore, factors other than pathologic stage influenced the natural history of localized ccRCC. Hence, integrated prognostic algorithms are necessary to better prognosis. As widely used prognosis models, UISS, SSIGN and Leibovich scores only focus on the features of tumor cells, but ignore the importance of tumor microenvironment in tumor development and progression. So it is reasonable that combine MUC3A expression with these models would improve prognostic stratification. Meanwhile, the nomogram constructed above offered a novel prognostic system for patients' clinical practice. In this sense, we advocate staining samples with MUC3A after surgery to intensify postoperative management. For instance, localized ccRCC patients with high MUC3A expression may have to choose neoadjuvant therapy and advanced follow-up after surgery.

Besides its meaningful prognostic value, the prospect of MUC3A in cancer diagnosis and treatment has also been paid more and more attention these years. Given the non-invasive and cost burden of serum biomarkers, abnormal mucin glycandetected in blood has been applied in certain malignancies though several clinically approved serodiagnostic tests [23]. For instance, the epitope of MUC1 named Cancer Antigen 27-29 (CA 27-29) was used to monitor breast cancer. Similar to an epitope of MUC16, Cancer Antigen 125 (CA-125) was used in the diagnosis of ovarian cancer. As similar to MUC1 and MUC16, MUC3A is also a membrane-associated mucin and maybe applied in serodiagnostic tests to diagnosis and monitor cancers. Given the current researches of mucins in treatment, MUC3A may win a place as well. There were several studies about anti-MUC-1 vaccines for cancers [24-27]. In phase III clinical trials for metastatic breast cancer patients, an anti-MUC-1 vaccine named sTn-KLH vaccine has been proven to induce humoral and cellular immune responses [24-26].

Although the clinical significance of MUC3A in localized ccRCC had been revealed, there are still several limitations in our study needing further discussion. Firstly, absence of an independent cohort for external validation to identify our findings. Secondly, we did not include patients with metastasis and MUC3A expression failed to stratify non–clear cell patients in our current study. The reason might be that few metastasis patients undertake surgery treatment and the specimen size of non-ccRCC is small. We would like to further analyze the prognostic effect of MUC3A in metastasis ccRCC, chromophobe RCC and papillary RCC separately in the future by enrolling adequate patients. Thirdly, radiologically indeterminate renal masses were increasing using percutaneous renal tumor biopsy to confirm histologic diagnosis. Thereby, we can further explore whether MUC3A could be a diagnostic marker in kidney biopsy samples. Fourthly, as a retrospective study, blood samples are not available from patients. Thus whether serum-based MUC3A level is associated with clinical outcomes is not clear. Further examination with blood samples is needed. Finally, the biological mechanism of MUC3A in localized ccRCC still needs further functional studies to elucidate.

In summary, our study demonstrated that MUC3A expression is a novel adverse independent biomarker in clinical outcomes of patients after nephrectomy with localized ccRCC. A prognostic nomogram integrating MUC3A expression and other factors may improve the management of prognostic stratification, personalizing postsurgical surveillance, and selecting patients for adjuvant therapy. Functional researches are necessary to reveal the molecular mechanisms of MUC3A in ccRCC and its role in diagnosis and therapeutic target.

## MATERIALS AND METHODS

### Patients

We retrospectively enrolled 384 consecutive patients with localized ccRCC (T1-3N0M0) who were treated with radical or partial nephrectomy between 2008 and 2009 at Zhongshan Hospital (Shanghai, China). Hospital's Ethics Committee has granted Ethical approval for this study, and informed consent has been signed by every patient. The inclusion criteria were: 1) cc RCC proved by histopathology, 2) patients without T4, N1 or M1 disease, 3) no history of other malignancies, 4) no neoadjuvant treatment, 5) no perioperative mortality, 6) no bilateral disease and familial RCC. Comprehensive clinicopathologic information was collected, including age at surgery, gender, histopathology, tumor size, TNM category, necrosis, lymphovascular invasion (LVI), Fuhrman grade, Eastern Cooperative Oncology Group performance status (ECOG-PS). Tumor stages were assigned by the TNM classification of 2010 American Joint Committee on Cancer [28]. The UISS, SSIGN and Leibovich prognostic models were used to stratify all patients, respectively. Follow-up was carried out biyearly for the first two years and yearly thereafter to collecting patients' physical examination, laboratory tests, MRI and CT scans. Recurrence was confirmed by radiology and histopathology as follows: 1) tumor relapse in operative field; 2) regional lymph nodes; 3) distant metastasis. Follow-up was finished on October 2013. Recurrence-free survival (RFS) and overall survival (OS) were calculated the interval from surgery to recurrence or death from all patients, respectively.

### Immunohistochemistry

Tissue microarrays (TMA) were constructed and stained by immunohistochemical at once as mentioned previously [29]. Primary anti-MUC3A-antibody-ab199260 (diluted 1:50; Abcam, Cambridge, UK) was applied for immunohistochemistry staining. The staining intensity and percentage of positive cells for immunohistochemical analysis was evaluated independently by two pathologists unaware of the patient information. To avoid the interobserver variability, the mean value of H-scores was adapted for further analysis. A semiquantitative score was calculated by multiplication with the staining intensities (0, negative; 1, weak; 2, moderate; 3, strong) and the distribution regions (0–100 %), ranging from 0 to 300.

### Statistical analysis

The patients were optimally separated into low and high expression subgroup by X-tile software. Chi-square test, Mann-Whitney *U* tests for categorical variable and student *t* test for continuous variable are used to analysis the correlation between characteristics variables and MUC3A expression. Survival curves were based on Kaplan-Meier method and assessed by log-rank test. The univariate and multivariate Cox regression analysis were performed for prognostic factors. The accuracy of the prognostic models was evaluated by Harrell's concordance index (*c*-index). Furthermore, a nomogram for OS and RFS based on multivariable analysis was constructed and calibrated as previously described [30]. In this study, all statistical tests' *P* values were two-sided, and 0.05 was considered a significant level of the *P* values. Date analysis was conducted by X-tile software v3.6.1 (Yale University, New Haven, CT, USA), MedCalc Software (version 15.2.2; MedCalc, Mariakerke, Belgium), IBM SPSS Statistics 19.0 (IBM Corp, Armonk, New York), Stata 14.0 (StataCorp, College Station, TX) and R software 3.2.3 (‘rms’ package, R Foundation for Statistical Computing, Vienna, Austria).

## SUPPLEMENTARY MATERIALS FIGURES


