# Supplementary material for: Increased expression of MUC3A is associated with poor prognosis in localized clear-cell renal cell carcinoma
Source: Oncotarget. 2016 Jun 27;7(31):50017–26. doi: 10.18632/oncotarget.10312 (PMC5226565; doi:10.18632/oncotarget.10312)
Supplement: Supplementary file 1 [file oncotarget-07-50017-s001.pdf]

Increased expression of MUC3A is associated with poor prognosis in localized clear-cell renal cell carcinoma

SUPPLEMENTARY FIGURES

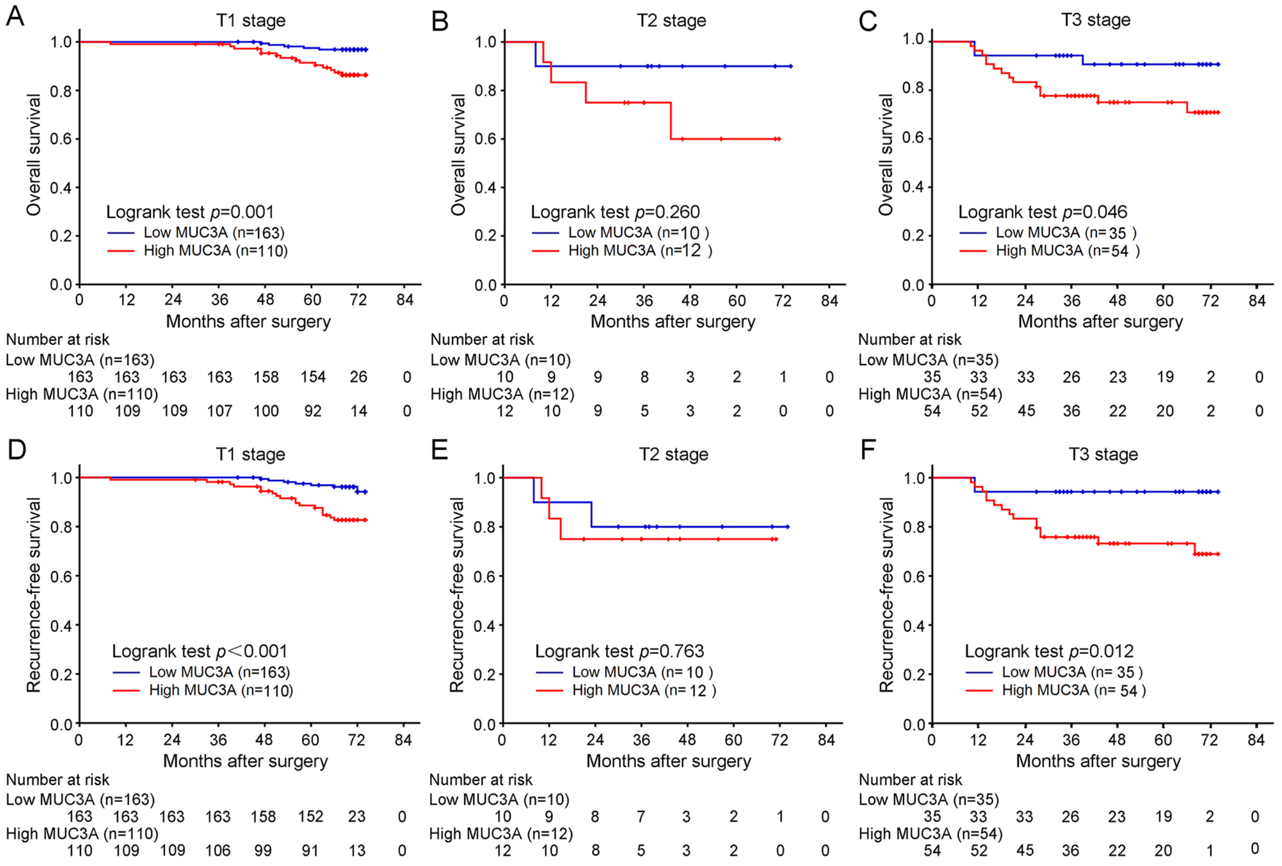

Supplementary Figure S1: Overall survival (OS) and Recurrence-free survival (RFS) analysis of patients with localized ccRCC based on MUC3A expression in pT subgroups. Kaplan-Meier analyses for OS **A.** and RFS **D.** of localized ccRCC patients according to MUC3A expression in T1 stage. Kaplan-Meier analyses for OS and RFS of localized ccRCC patients according to MUC3A expression in T2 stage OS **B.** and RFS **E.** Kaplan-Meier analyses for OS and RFS of localized ccRCC patients according to MUC3A expression in T3 stage OS **C.** and RFS **F.** *P* value was calculated by log-rank test.

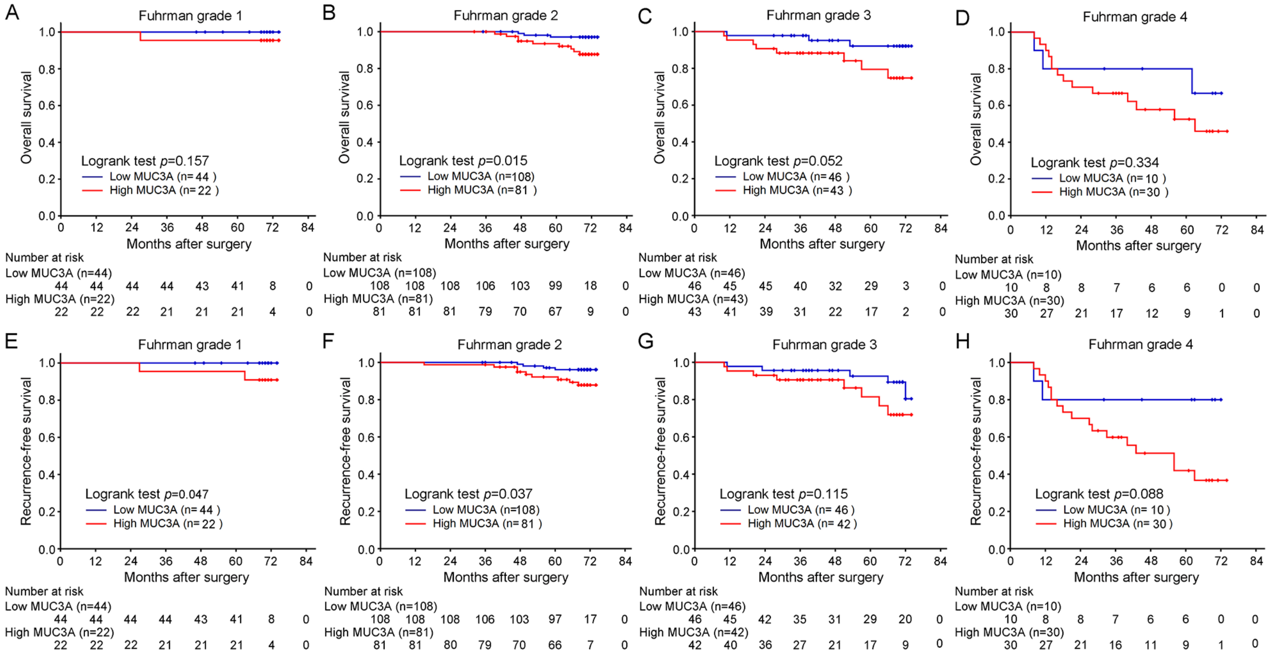

Supplementary Figure S2: Overall survival (OS) and Recurrence-free survival (RFS) analysis of patients with localized ccRCC based on MUC3A expression in Fuhrman grade subgroups. Kaplan-Meier analyses for OS and RFS of localized ccRCC patients according to MUC3A expression in Fuhrman grade 1 OS **A.** and RFS **E.** Kaplan-Meier analyses for OS and RFS of localized ccRCC patients according to MUC3A expression in Fuhrman grade 2 OS **B.** and RFS **F.** Kaplan-Meier analyses for OS and RFS of localized ccRCC patients according to MUC3A expression in Fuhrman grade 3 OS **C.** and RFS **G.** Kaplan-Meier analyses for OS and RFS of localized ccRCC patients according to MUC3A expression in Fuhrman grade 4 OS **D.** and RFS **H.** *P* value was calculated by log-rank test.

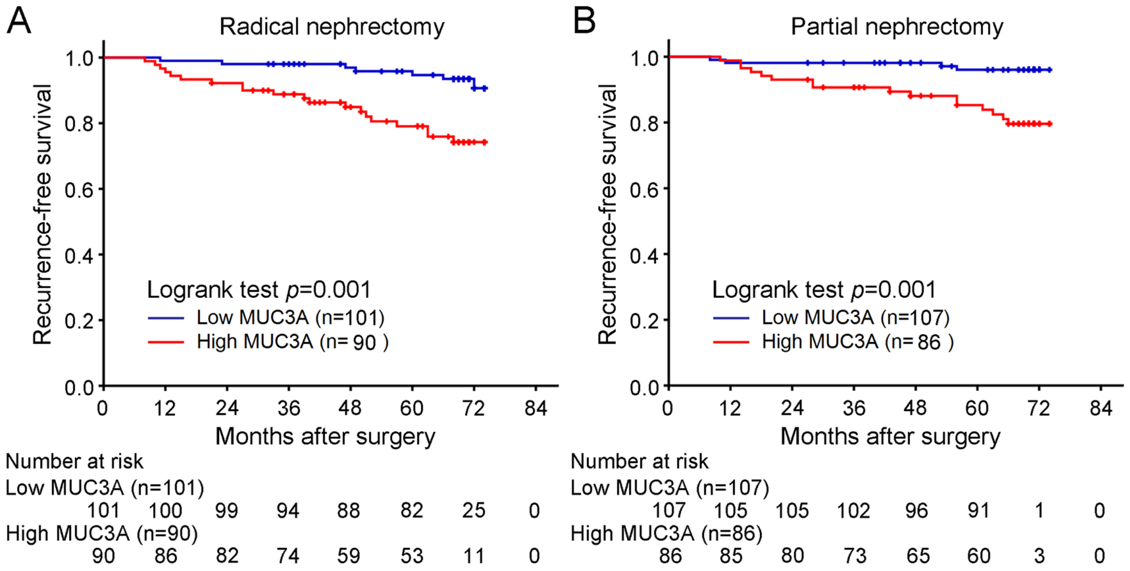

Supplementary Figure S3: Comparison of RFS according to MUC3A expression in patients underwent radical nephrectomy and partical nephrectomy, respectively. Kaplan-Meier analysis of RFS in radical nephrectomy group **A.** partial nephrectomy group **B.** P value was calculated by log-rank test.
